# Association of advanced lung cancer inflammation index with the prevalence and all-cause mortality in patients with lung cancer

**DOI:** 10.1097/MD.0000000000047172

**Published:** 2026-01-23

**Authors:** Huijuan Cheng, Weikai Lin, Peilin You, Cuili Lin, Zhen Zhang, Weilan Lin, Shun Chen, Feng Lu

**Affiliations:** a The Second Affiliated Hospital of Fujian University of Traditional Chinese Medicine, Fuzhou, Fujian, China; b Fujian University of Traditional Chinese Medicine, Fuzhou, Fujian, China; c Clinical Research Center for Integrative Medicine on Early Lung Cancer Diagnosis and Treatment of Fujian Province, Fuzhou, Fujian, China.

**Keywords:** advanced lung cancer inflammation index, lung cancer, NHANES

## Abstract

The advanced lung cancer inflammation index (ALI) has been proposed as a potential prognostic biomarker in lung cancer. However, evidence linking ALI to the prevalence of lung cancer and all-cause mortality remains limited. This study aimed to investigate the association between ALI and both the risk of lung cancer and all-cause mortality among individuals diagnosed with the disease. Data were obtained from 38,213 adults aged ≥ 20 years who participated in the 1999 to 2018 National Health and Nutrition Examination Survey. Cross-sectional analyses were performed to evaluate the association between ALI and lung cancer prevalence, while longitudinal analyses assessed the relationship between ALI and all-cause mortality among lung cancer patients. Multivariable logistic regression and Cox proportional hazards models were used, adjusting for relevant covariates. Multiple imputation was applied in sensitivity analyses to address missing data. Higher ALI levels were significantly associated with a lower risk of lung cancer. In the fully adjusted model, participants in the highest ALI quartile (Q4) had a 58% reduced risk compared to those in the lowest quartile (Q1) (OR = 0.42, 95% CI: 0.20–0.87, *P* = .019). Additionally, elevated ALI was linked to reduced all-cause mortality among individuals diagnosed with lung cancer. ALI may help stratify lung cancer risk in the general population and may be associated with overall survival outcomes among patients with the disease. Incorporating ALI into clinical risk assessments may aid in the development of more personalized treatment strategies. Prospective studies are needed to validate these findings and elucidate their underlying mechanisms.

## 1. Introduction

Lung cancer remains one of the most frequently diagnosed malignancies and the leading cause of cancer-related death worldwide,^[1,2]^ accounting for approximately 1.8 million deaths annually.^[3]^ In the United States, the five-year survival rate for lung cancer remains below 21%, with more than 57% of cases diagnosed at an advanced stage.^[4]^ Non-small-cell lung cancer, the predominant histologic subtype, accounts for nearly 85% of all cases and encompasses adenocarcinoma, squamous cell carcinoma, and large cell carcinoma.^[5,6]^ In addition to established prognostic factors such as tumor stage, histological subtype, and molecular alterations, systemic inflammation has been increasingly recognized as a key biological mechanism contributing to tumor progression, therapeutic resistance, and cancer-associated cachexia.^[7,8]^ This inflammatory state is often assessed using hematologic parameters such as the neutrophil-to-lymphocyte ratio (NLR) and platelet-to-lymphocyte ratio, which reflect the complex interactions between the host immune system and the tumor microenvironment. Elevated values of these markers have been consistently associated with poor clinical outcomes.^[9,10]^ Given the limitations of using single biomarkers, the advanced lung cancer inflammation index (ALI) was developed as a composite prognostic score that integrates nutritional status, reflected by body mass index (BMI) and serum albumin, with systemic inflammation, measured by NLR.^[11]^ Preliminary validation studies have consistently shown that lower pretreatment ALI levels are associated with worse overall survival (OS) and progression-free survival among patients with lung cancer across various study populations. These findings support its potential use as an independent prognostic indicator.^[12–14]^ From a biological standpoint, a low ALI reflects a state characterized by increased systemic inflammation and compromised nutritional condition. Both of these factors are known to contribute to tumor development, angiogenesis, immune escape, and metabolic dysregulation.^[15,16]^

Nevertheless, several important knowledge gaps remain to be addressed. First, most of the available evidence is derived from relatively small-scale, single-center studies or populations undergoing specific types of treatment. This limits the generalizability and external validity of previous findings.^[13,17]^ Second, although the association between ALI and lung cancer–related mortality has been supported by prior research, its relationship with lung cancer prevalence has not been sufficiently explored. Clarifying whether ALI can serve as a marker of lung cancer susceptibility may have significant implications for prevention and early detection. Third, the prognostic value of ALI across different demographic and clinical subgroups within large population-based samples remains unclear and requires rigorous investigation.

Accordingly, this study aimed to examine the association between ALI and the prevalence of lung cancer, as well as its relationship with all-cause mortality among patients with lung cancer, based on data from the National Health and Nutrition Examination Survey (NHANES).

## 2. Methods

### 2.1. Study population

NHANES is an ongoing, nationally representative cross-sectional study designed to evaluate the health and nutritional status of the non-institutionalized U.S. population. It integrates structured interviews, physical examinations, and laboratory tests. A complex, multistage, stratified probability sampling design was employed to ensure national representativeness.^[18]^ The study protocol was approved by the Research Ethics Review Board of the National Center For Health Statistics, and all participants provided written informed consent. Participants from the 1999 to 2018 NHANES cycles were included in the present study, as the linked mortality follow-up data are available through December 31, 2019, corresponding to these survey years. Inclusion of later cycles would lead to incomplete mortality ascertainment because deaths occurring after the linkage cutoff have not yet been released. NHANES data are publicly available at https://www.cdc.gov/nchs/nhanes/ (accessed on June 2, 2025).

This study included data from adult participants (aged ≥ 20 years) in NHANES during the 1999 to 2018 cycles. These cycles were selected based on the availability of variables relevant to the study objectives. Participants were excluded if they had missing information on laboratory measurements, lung cancer status, covariates, or mortality data.

### 2.2. Assessment of ALI

ALI was evaluated as a composite index reflecting both nutritional status and systemic inflammation. It was calculated using the following formula: ALI = BMI (kg/m^2^) × serum albumin (g/dL)/NLR, where NLR was derived by dividing the absolute neutrophil count by the absolute lymphocyte count.^[11]^ BMI was calculated from measured height and weight, serum albumin was obtained from blood samples, and NLR was derived from complete blood count data. Higher ALI values indicate better nutritional reserves and lower systemic inflammatory burden. For stratified analyses, ALI was categorized into 4 groups based on quartile distribution: very low (<46.0), low (46.0–63.1), moderate (63.2–85.8), and high (>85.8). This classification enabled a systematic comparison of lung cancer risk across different ALI levels, thereby improving the robustness and interpretability of the results.

### 2.3. Assessment of outcome

Lung cancer status was determined based on responses to a questionnaire item asking, “Have you ever been told by a doctor or other health professional that you had cancer or a malignancy of any kind?” Participants who answered “Yes” were subsequently asked to specify the type of cancer. Individuals who reported lung cancer were classified as having a lung cancer diagnosis.

For the longitudinal analysis, mortality status was ascertained by linking NHANES participants to the National Death Index records through December 31, 2019. All-cause mortality was defined as death from any cause. Mortality status and follow-up time were calculated from the date of the NHANES examination to the date of death or the end of the follow-up period, whichever came first.

### 2.4. Covariates definitions

Based on previously published studies and clinical relevance, potential covariates included age, sex, race/ethnicity, educational attainment, BMI, smoking status, alcohol consumption, diabetes, and hypertension.^[19,20]^ Educational attainment was categorized into 3 levels according to years of schooling: <9 years, 9 to 12 years, and >12 years. Smoking status was classified as never, former, or current smoker, according to the established definitions in prior research.^[21]^ Similarly, alcohol consumption was categorized as never, former, or current drinker.^[21]^ Diabetes was defined according to any of the following criteria: a self-reported physician diagnosis; a random plasma glucose or 2-hour oral glucose tolerance test result ≥ 11.1 mmol/L; a fasting plasma glucose ≥ 7.0 mmol/L; glycated hemoglobin (HbA1c) > 6.5%; or current use of insulin or antidiabetic medications.^[22]^ Hypertension was defined as any of the following conditions: self-reported physician diagnosis, current use of antihypertensive medications, or a mean systolic blood pressure ≥ 140 mm Hg or mean diastolic blood pressure ≥ 90 mm Hg based on the average of 3 standardized blood pressure measurements collected during the NHANES physical examination.^[23]^

### 2.5. Study design

This study adopted a two-stage design using data from NHANES to investigate the association between ALI, lung cancer risk, and all-cause mortality among individuals diagnosed with lung cancer. In the first stage, a cross-sectional analysis was conducted to evaluate the association between ALI and the prevalence of lung cancer at baseline. In the second stage, a longitudinal analysis was performed to assess the relationship between ALI and all-cause mortality among participants with confirmed lung cancer. Mortality data were obtained from the NHANES Public Use Linked Mortality File, enabling survival analysis over the follow-up period.

### 2.6. Statistical analysis

All statistical procedures were conducted in accordance with the analytical guidelines provided by NHANES, with careful consideration of the complex multistage sampling design and appropriate sample weights.^[18]^ The sample weights were calculated as follows: for the 1999–2002 survey cycles, the weight was computed as 2/10 of the 4-year MEC sample weight; for the 2003 to 2018 cycles, it was calculated as 1/10 of the 2-year MEC sample weight. Categorical variables are presented as unweighted frequencies with weighted percentages, while continuous variables are expressed as means with standard errors. Group comparisons were performed using linear regression models for continuous variables and chi-square tests for categorical variables.

To explore the association between ALI and lung cancer prevalence, multivariable logistic regression models were applied to estimate odds ratios (OR) and 95% confidence intervals (CIs). To assess the relationship between ALI and all-cause mortality among patients with lung cancer, multivariable Cox proportional hazards regression models were used to calculate hazard ratios (HRs) and corresponding 95% CIs. Two models were constructed for adjustment. Model 1 included age, sex, and race/ethnicity. Model 2 further adjusted for educational attainment, BMI, smoking status, alcohol consumption, diabetes, and hypertension. To examine the distribution of ALI across participants, values were categorized into quartiles based on the 25th, 50th (median), and 75th percentiles of the study population. Dose–response relationships between ALI and both lung cancer risk and all-cause mortality were evaluated using restricted cubic spline (RCS) regression models. In addition, a threshold effect analysis was conducted to identify potential inflection points in the associations between ALI and both lung cancer risk and all-cause mortality among patients with lung cancer. Kaplan–Meier survival curves were generated to visualize survival outcomes among patients with lung cancer.

Subgroup analyses were conducted using stratified multivariable logistic regression models according to sex (male vs female), age (<45 vs ≥45 years), race/ethnicity (non-Hispanic White vs others), BMI (<25 vs ≥25 kg/m^2^), smoking status (never vs former/current), and alcohol use (never vs former/current). Missing covariate data were addressed using multiple imputation with 5 replications to reduce bias and maintain statistical power. All statistical analyses were performed using R software (version 4.2.2) and Free Statistics software (version 2.2). Two-sided *P*-values < .05 were considered statistically significant. Bootstrapping and penalized regression were not applied because the complex multistage sampling design of NHANES requires specialized resampling methods that properly account for survey weights, clustering, and stratification. A parsimonious modeling approach was adopted to minimize overfitting and ensure robust estimates.

## 3. Results

### 3.1. Study population

**Figure 1.**
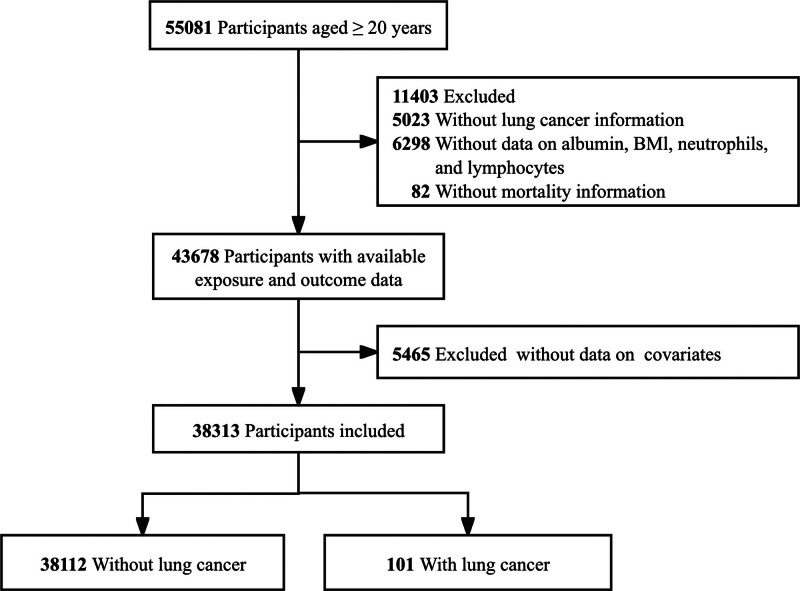
Flowchart of participant selection from the US NHANES. NHANES = National Health and Nutrition Examination Survey.

Among the 55,081 adult participants aged 20 years and older, a total of 16,868 individuals were excluded due to missing laboratory data (n = 6298), unavailable self-reported lung cancer information (n = 5023), missing covariate data (n = 5465), or incomplete mortality records (n = 83). Consequently, 38,213 participants were included in the final analysis, as illustrated in Figure 1.

### 3.2. Baseline characteristics

Table 1 summarizes the baseline characteristics of the 38,213 participants included in the study, among whom 101 individuals (0.26%) were diagnosed with lung cancer. The mean age of the study population was 45.73 (±0.20) years, with males accounting for 19,330 (50.2%). Compared to participants without lung cancer, those with lung cancer were significantly older (66.37 ± 1.3 vs 45.68 ± 0.2 years) and exhibited higher prevalences of hypertension (61.6% vs 35.1%) and diabetes (35.0% vs 11.9%). Significant differences were also observed between the 2 groups in terms of race/ethnicity (non-Hispanic White: 84.7% vs 68.2%), smoking status (former smokers: 71.9% vs 23.7%), and alcohol consumption history (former drinkers: 41.1% vs 13.8%) (all *P* < .001). Educational attainment varied as well, with a lower proportion of participants in the lung cancer group having completed more than 12 years of education (41.6% vs 59.0%). No significant differences were found in sex distribution or body mass index (BMI: 28.23 ± 1.0 vs 28.78 ± 0.1 kg/m^2^) between groups.

**Table 1:** Baseline characteristics of participants by lung cancer status. Data are presented as unweighted number (weighted percentage) for categorical variables and mean (SE) for continuous variables.

Characteristic	Overall (n = 38,213)	No lung cancer (n = 38,112)	Prevalent lung cancer (n = 101)	*P*-value
Age, mean (SE), years	45.73 (0.2)	45.68 (0.2)	66.37 (1.3)	<.001
Sex				
Male	19,330 (50.2)	19,269 (50.2)	61 (51.1)	.88
Female	18,883 (49.8)	18,843 (49.8)	40 (48.9)	
Education level, yr				
<9	4508 (5.6)	4496 (5.6)	12 (11.2)	.018
9–12	14,552 (35.5)	14,505 (35.4)	47 (47.3)	
>12	19,153 (58.9)	19,111 (59.0)	42 (41.6)	
Race/ethnicity				
Non-Hispanic White	16,543 (68.3)	16,475 (68.2)	68 (84.7)	<.001
Non-Hispanic Black	7877 (10.8)	7858 (10.8)	19 (9.0)	
Mexican American	7136 (8.6)	7135 (8.6)	1 (0.20)	
Other	6657 (12.4)	6644 (12.4)	13 (6.1)	
Smoking status				
Never	20,792 (54.2)	20,780 (54.3)	12 (11.9)	<.001
Former	9098 (23.8)	9027 (23.7)	71 (71.90)	
Current	8323 (22.0)	8305 (22.0)	18 (17.1)	
Alcohol drinking status				
Never	5473 (11.3)	5464 (11.3)	9 (9.0)	<.001
Former	6441 (13.9)	6399 (13.8)	42 (41.1)	
Current	26,299 (74.8)	26,249 (74.9)	50 (49.9)	
Body mass index, kg/m^2^	28.78 (0.1)	28.78 (0.1)	28.23 (1.0)	.33
Hypertension	15,500 (35.1)	15,434 (35.1)	66 (61.6)	<.001
Diabetes	6360 (12.0)	6327 (11.9)	33 (35.0)	<.001

SE = standard error.

### 3.3. Association between ALI and lung cancer

Multivariable regression analysis (Table 2) revealed that higher ALI quartiles were significantly associated with a lower prevalence of lung cancer. In the fully adjusted model (Model 2), controlling for demographic, lifestyle, and clinical factors, the risk of lung cancer progressively decreased across increasing ALI quartiles compared with the lowest quartile (Q1): Q2 (OR = 0.17; 95% CI: 0.09–0.36; *P* < .001), Q3 (OR = 0.34; 95% CI: 0.14–0.83; *P* = .018), and Q4 (OR = 0.42; 95% CI: 0.20–0.87; *P* = .019).

**Table 2:** Association between advanced lung cancer inflammation index and the risk of developing lung cancer.

	Crude model		Model 1		Model 2	
	OR (95% CI)	*P*-value	OR (95% CI)	*P*-value	OR (95% CI)	*P*-value
ALI						
Q1 (<46.0)	Reference		Reference		Reference	
Q2 (46.0–63.1)	0.12 (0.06–0.24)	<.001	0.17 (0.08–0.34)	<.001	0.17 (0.09–0.36)	<.001
Q3 (63.2–85.8)	0.21 (0.10–0.45)	<.001	0.32 (0.15–0.70)	.004	0.34 (0.14–0.83)	.018
Q4 (>85.8)	0.25 (0.13–0.47)	<.001	0.41 (0.22–0.77)	.006	0.42 (0.20–0.87)	.019

Crude model: nonadjusted model; Model 1: adjusted for age, sex, race; Model 2: adjusted for covariates of Model 1, and smoking status, drinking status, education level, body mass index, diabetes, and hypertension.

ALI = advanced lung cancer inflammation index, CI = confidence interval, OR = odds ratio, Q = quartile.

**Figure 2.**
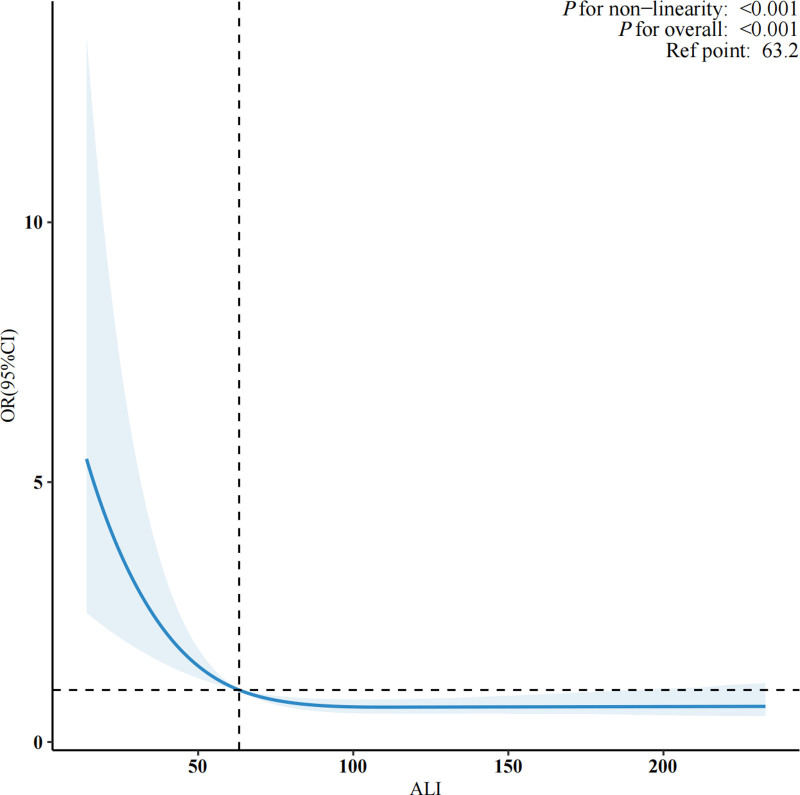
Dose–response relationship between the ALI and the risk of lung cancer, assessed by restricted cubic spline regression. ALI = advanced lung cancer inflammation index.

The RCS analysis (Fig. 2) demonstrated a nonlinear inverse association between ALI and lung cancer risk. This relationship was further evaluated using a threshold effect analysis (Table 3), which revealed that the association was statistically significant only when ALI was below 54.6 (OR = 0.94; 95% CI: 0.92–0.97; *P* < .001). In contrast, when ALI was equal to or >54.6, no significant association was observed (OR = 0.99; 95% CI: 0.96–1.01; *P* = .220). These findings suggest that the protective effect of ALI against lung cancer risk is predominantly confined to values below this exploratory threshold.

**Table 3:** Threshold effect analysis of the relationship of advanced lung cancer inflammation index with lung cancer.

ALI	Adjusted model	
	OR (95% CI)	*P*-value
<54.6	0.94 (0.92–0.97)	<.001
≥54.6	0.99 (0.96–1.01)	.22

ALI = advanced lung cancer inflammation index, CI = confidence interval, OR = odds ratio.

### 3.4. ALI and all-cause mortality in lung cancer patients

Multivariable regression analysis (Table 4) demonstrated that higher ALI quartiles were significantly associated with lower all-cause mortality among patients with lung cancer. In the fully adjusted model (Model 2), individuals in the highest ALI quartile (Q4) exhibited a significantly lower risk of death compared to those in the lowest quartile (Q1) (HR = 0.19; 95% CI: 0.05–0.71; *P* = .013), corresponding to an 81% reduction in mortality risk.

**Table 4:** Association between advanced lung cancer inflammation index and the risk of all-cause mortality among lung cancer patients.

	Crude model		Model 1		Model 2	
	HR (95% CI)	*P*-value	HR (95% CI)	*P*-value	HR (95% CI)	*P*-value
ALI						
Q1 (<46.0)	Reference		Reference		Reference	
Q2 (46.0–63.1)	0.37 (0.16–0.85)	.018	0.52 (0.22–1.21)	.13	0.54 (0.18–1.63)	.277
Q3 (63.2–85.8)	0.42 (0.15–1.20)	.105	0.43 (0.16–1.18)	.101	0.31 (0.08–1.20)	.09
Q4 (>85.8)	0.11 (0.03–0.36)	<.001	0.19 (0.06–0.61)	.005	0.19 (0.05–0.71)	.013

Crude model: nonadjusted model; Model 1: adjusted for age, sex, race; Model 2: adjusted for covariates of Model 1, and smoking status, drinking status, education level, body mass index, diabetes, and hypertension.

ALI = advanced lung cancer inflammation index, CI = confidence interval, HR = hazard ratio, Q = quartile.

**Figure 3.**
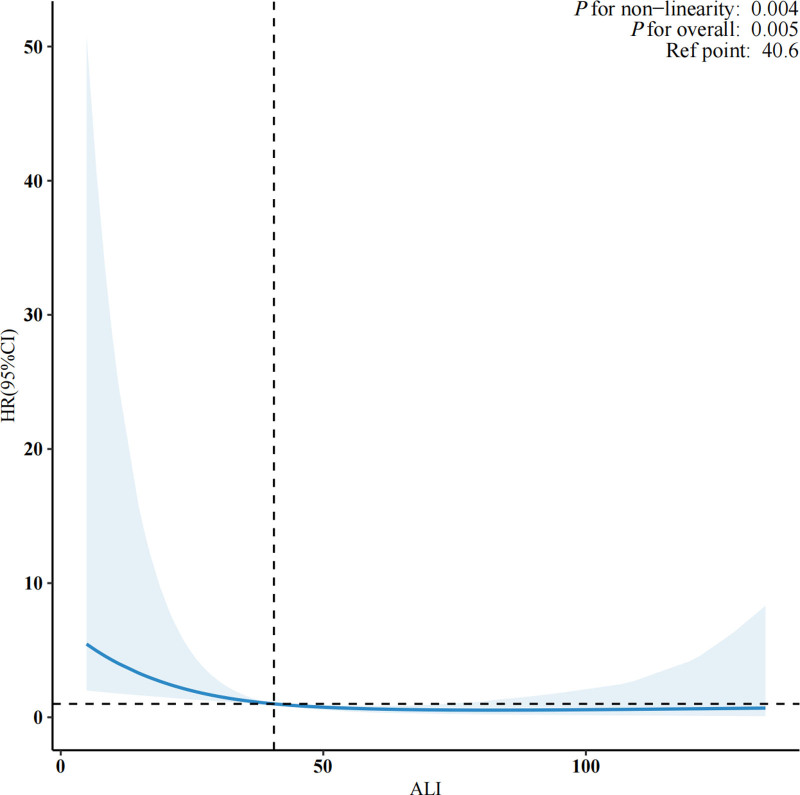
Dose–response relationship between the ALI and all-cause mortality among patients with lung cancer, assessed by restricted cubic spline regression. ALI = advanced lung cancer inflammation index.

**Figure 4.**
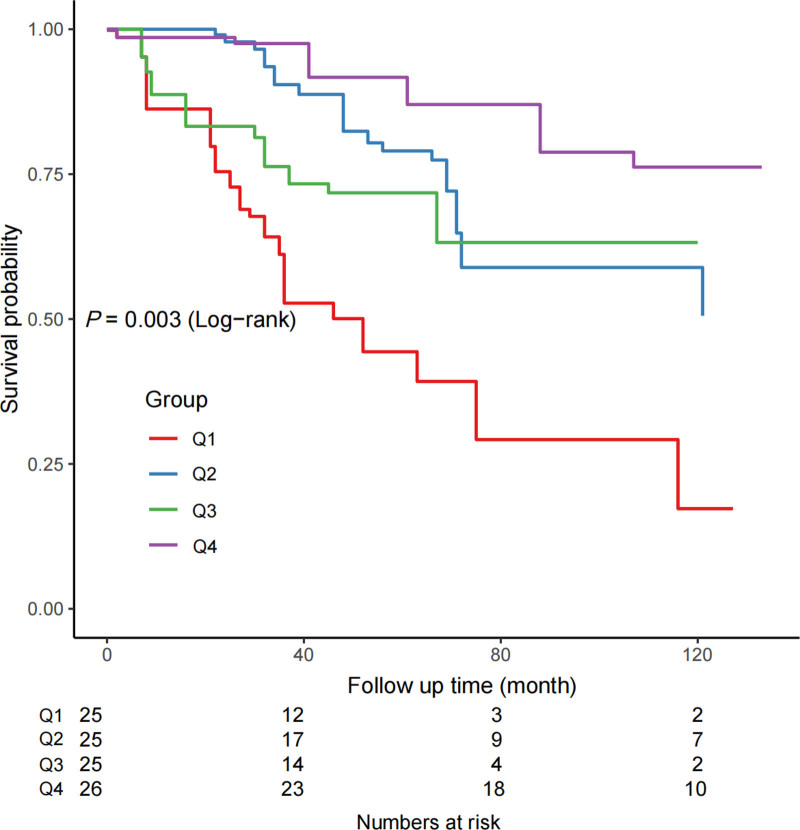
Kaplan–Meier survival curves for all-cause mortality according to the ALI. ALI = advanced lung cancer inflammation index.

RCS analysis (Fig. 3) confirmed a nonlinear dose–response relationship between ALI and mortality (*P* for nonlinearity = .004). Threshold analysis (Table 5) indicated that the inverse association was significant only when ALI was <33.7 (HR = 0.91; 95% CI: 0.84–0.99; *P* = .03), while no significant effect was observed for ALI ≥ 33.7 (HR = 0.99; 95% CI: 0.97–1.02; *P* = .70). These findings suggest that the survival benefit of higher ALI is primarily concentrated below this exploratory threshold. Kaplan–Meier survival curves (Fig. 4) further corroborated these findings, showing significantly higher cumulative survival in the Q4 group compared to lower quartiles throughout follow-up (log-rank *P* < .001), with a 40% absolute survival advantage at 60 months.

**Table 5:** Threshold effect analysis of the relationship of advanced lung cancer inflammation index with the risk of all-cause mortality among lung cancer patients.

ALI	Adjusted model	
	HR (95% CI)	*P* value
<33.7	0.91 (0.84–0.99)	.03
≥33.7	0.99 (0.97–1.02)	.7

ALI = advanced lung cancer inflammation index, CI = confidence interval, OR = odds ratio.

### 3.5. Subgroup analyses

**Figure 5.**
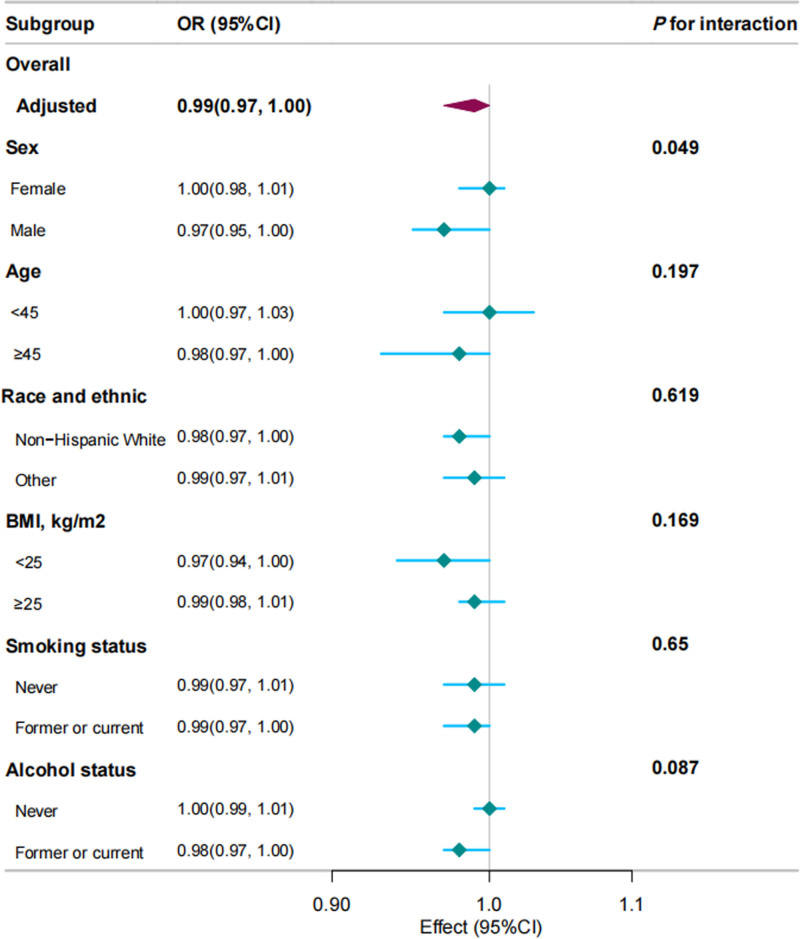
Stratified analyses of the associations between the ALI and the risk of developing lung cancer. ALI = advanced lung cancer inflammation index.

Figure 5 presents the results of the stratified analysis. Significant associations between ALI and lung cancer prevalence were observed in specific subgroups, including males (OR = 0.97; 95% CI: 0.95–1.00), individuals aged ≥ 45 years (OR = 0.98; 95% CI: 0.97–1.00), non-Hispanic Whites (OR = 0.98; 95% CI: 0.97–1.00), participants with BMI < 25 kg/m^2^ (OR = 0.97; 95% CI: 0.94–1.00), former or current smokers (OR = 0.99; 95% CI: 0.97–1.00), and former or current drinkers (OR = 0.98; 95% CI: 0.97–1.00). In contrast, no significant associations were found among females, participants younger than 45 years, those from other racial/ethnic backgrounds, individuals with a BMI ≥ 25 kg/m^2^, never smokers, or those who had never consumed alcohol.

### 3.6. Sensitivity analysis

Table 6 shows the results of the sensitivity analysis based on multiple imputation with 5 replications to account for missing covariate data. The association between ALI and lung cancer remained consistent across all models. In the fully adjusted model, individuals in higher ALI quartiles had significantly lower odds of lung cancer (Model 2: Q2 = 0.19, Q3 = 0.30, Q4 = 0.40; all *P* < .013).

**Table 6:** Sensitivity analysis after multiple imputation of missing data: association between advanced lung cancer inflammation index and the risk of developing lung cancer.

	Crude model		Model 1		Model 2	
	OR (95% CI)	*P*-value	OR (95% CI)	*P*-value	OR (95% CI)	*P*-value
ALI						
Q1 (<46.0)	Reference		Reference		Reference	
Q2 (46.0–63.1)	0.14 (0.07–0.26)	<.001	0.18 (0.10–0.35)	<.001	0.19 (0.10–0.37)	<.001
Q3 (63.2–85.8)	0.18 (0.09–0.39)	<.001	0.28 (0.13–0.59)	.001	0.3 (0.12–0.70)	.006
Q4 (>85.8)	0.24 (0.13–0.44)	<.001	0.38 (0.20–0.72)	.003	0.4 (0.19–0.82)	.013

Crude model: unadjusted model; Model 1: adjusted for age, sex and race; Model 2: adjusted for covariates of Model 1, and smoking status, drinking status, education level, body mass index, diabetes, and hypertension.

ALI = advanced lung cancer inflammation index, CI = confidence interval, OR = odds ratio, Q = quartile.

## 4. Discussion

Our results indicate that higher levels of ALI are significantly inversely associated with both the prevalence of lung cancer and all-cause mortality among patients with lung cancer. These associations remained robust across various subgroup and sensitivity analyses.

These findings further reinforces the pivotal role of systemic inflammation and nutritional status in both the development and prognosis of cancer. In recent years, accumulating evidence has consistently shown that low ALI is strongly associated with unfavorable outcomes in lung cancer patients, underscoring its potential utility as an independent prognostic biomarker. For example, a cohort study conducted in Thailand involving 109 metastatic Non-small cell lung cancer patients receiving first-line chemotherapy found that lower ALI was associated with shorter OS (HR = 1.42; 95% CI: 0.67–3.01).^[24]^ Another study identified ALI ≥ 70.06 as a favorable prognostic factor for OS in non-small cell lung cancer patients (HR = 0.443; 95% CI: 0.231–0.850).^[25]^ Similarly, Song et al reported that lower ALI was linked to an increased risk of lung cancer–specific mortality in a multi-center cohort (HR = 1.30; 95% CI: 1.13–1.49).^[26]^ Moreover, Horstman et al extended these findings to patients treated with immune checkpoint inhibitors, demonstrating that higher ALI levels were significantly associated with prolonged survival (HR = 3.09; 95% CI: 2.36–4.06).^[12]^ Our findings are consistent with previous studies, further supporting the prognostic value of ALI in lung cancer.

From a clinical perspective, ALI is a practical and integrative marker that captures both nutritional status and systemic inflammation, showing significant promise for risk stratification. In this study, we identified distinct exploratory thresholds of ALI for predicting lung cancer prevalence (ALI < 54.6) and all-cause mortality (ALI < 33.7), both demonstrating clear dose–response relationships, thereby suggesting that ALI may serve as a prognostic and early-risk indicator. However, these thresholds should be interpreted as exploratory and require independent validation before clinical application, which underscores the potential utility of ALI for multi-outcome risk assessment while emphasizing caution in applying absolute cutoff values.

Building on these findings, ALI not only reflects systemic inflammatory and nutritional status but may also complement tissue-based immune markers, such as PD-L1 expression and tumor-infiltrating lymphocytes, thereby enhancing prognostic accuracy and improving patient stratification in clinical settings. Furthermore, ALI may provide insights into treatment response, particularly for systemic therapies such as chemotherapy and immune checkpoint inhibitors, where patients with higher ALI levels may experience better therapeutic outcomes. For individuals with lower ALI levels, nutritional support and anti-inflammatory interventions (such as COX-2 inhibitors) may offer preventive benefits. These strategies warrant further investigation in prospective studies to evaluate their impact on treatment response and survival, particularly given evidence that addressing inflammation and malnutrition may delay tumor progression.^[27,28]^

The biological rationale for this association is well established. ALI integrates nutritional indicators (BMI and albumin) with the systemic inflammation marker NLR, all of which are strongly associated with cancer development and progression. BMI reflects overall energy stores in the body and may serve as a useful surrogate marker for the early detection of cancer-related cachexia and muscle loss.^[29]^ Hypoalbuminemia often reflects malnutrition and reduced colloid osmotic pressure, which can impair tissue repair capacity and alter the tumor microenvironment, thereby promoting aggressive tumor behavior.^[30]^ An elevated NLR indicates a neutrophil-driven pro-inflammatory state, which may contribute to lung cancer progression through multiple mechanisms, including the promotion of tumor angiogenesis and immune evasion.^[31]^ Studies have demonstrated that high NLR is associated with increased secretion of pro-angiogenic factors such as vascular endothelial growth factor and interleukin-8, thereby facilitating neovascularization.^[32,33]^ At the same time, a relative reduction in lymphocyte count may impair immune surveillance, further supporting tumor development. Additionally, tumor-associated neutrophils can promote tumor cell proliferation and distant metastasis via activation of the JAK2/STAT3 signaling pathway. These neutrophils may also produce neutrophil extracellular traps, vascular endothelial growth factor, and matrix metalloproteinase-9, which further drive tumor angiogenesis.^[34]^

More importantly, our study provides several novel insights. First, we established a significant association between ALI and all-cause mortality in a nationally representative population, extending prior findings beyond cancer-specific mortality. This is particularly relevant given that all-cause mortality encompasses cardiovascular, metabolic, and infection-related deaths,^[35]^ which may be driven by chronic inflammation and malnutrition, thereby addressing limitations of earlier studies.^[24,25]^ Second, we offer novel evidence that lower ALI levels are linked to an elevated risk of developing lung cancer – an association that remains underexplored in the existing literature. This may suggest that a low ALI reflects a pro-carcinogenic state, potentially promoting tumor initiation through mechanisms such as chronic inflammation–induced genomic instability and impaired DNA repair.^[36]^ A clear dose–response relationship was observed, accompanied by a threshold effect, indicating that the inverse association became statistically significant only when ALI was below 54.6. Third, subgroup analyses revealed that the predictive value of ALI for lung cancer prevalence was more pronounced in specific populations, including males, individuals aged 45 years or older, non-Hispanic Whites, and those with lower BMI. While these findings require further validation, they suggest that the prognostic relevance of ALI may be modulated by demographic and metabolic backgrounds.

Although ALI was measured prior to mortality events and is supported by plausible biological mechanisms, the possibility of residual confounding from unmeasured factors, such as environmental exposures or genetic predispositions, cannot be excluded. Second, the diagnosis of lung cancer was based on self-reported questionnaire data, which may be subject to recall bias and misclassification. Nevertheless, several validation studies have demonstrated that NHANES self-reported cancer data are generally reliable when compared with medical records or cancer registry information, supporting their applicability in epidemiological analyses.^[37–39]^ Third, although smoking status was adjusted for, residual confounding due to unmeasured aspects such as smoking intensity and duration may still influence the results. Fourth, dynamic changes in ALI following cancer diagnosis, which may be affected by treatment or disease progression, were not captured in this analysis.

Future research should focus on: prospective validation of ALI cutoff values across diverse populations; investigation of whether modification of individual ALI components (e.g., albumin supplementation, NLR reduction) influences cancer risk and treatment response; integration of ALI with molecular and tissue-based immune biomarkers, such as PD-L1 and EGFR, to develop multidimensional prognostic models; and implementation of interventional trials to assess the effectiveness and feasibility of ALI-guided treatment strategies in high-risk populations, particularly in the context of systemic therapies and immunotherapy.

## 5. Conclusion

This study demonstrates that higher ALI levels are significantly associated with a lower prevalence of lung cancer and reduced all-cause mortality, with both associations exhibiting a nonlinear relationship. ALI may serve as a simple and effective risk assessment tool to identify high-risk individuals and inform personalized management strategies. Prospective studies are warranted to validate these findings and explore the underlying biological mechanisms.

## Author contributions

**Data curation:** Weikai Lin, Peilin You, Cuili Lin, Shun Chen.

**Visualization:** Zhen Zhang.

**Writing – original draft:** Huijuan Cheng.

**Writing – review & editing:** Weilan Lin, Feng Lu.
